# Responding to the deaf in disasters: establishing the need for systematic training for state-level emergency management agencies and community organizations

**DOI:** 10.1186/1472-6963-13-84

**Published:** 2013-03-07

**Authors:** Alina Engelman, Susan L Ivey, Winston Tseng, Donna Dahrouge, Jim Brune, Linda Neuhauser

**Affiliations:** 1 Health Research for Action, School of Public Health, University of California, Berkeley, 2140 Shattuck Avenue, 10th Floor, Berkeley, CA, 94704, USA; 2 Deaf Counseling, Advocacy and Referral Agency (DCARA), 14895 East 14th Street, Suite 200, San Leandro, CA, 94578-2926, USA

**Keywords:** All-hazards, Deaf, First responders, Emergency preparedness, Training

## Abstract

**Background:**

Deaf and hard-of-hearing (Deaf/HH) individuals have been underserved before and during emergencies. This paper will assess Deaf/HH related emergency preparedness training needs for state emergency management agencies and deaf-serving community-based organizations (CBOs).

**Methods:**

Four approaches were used: 1) a literature review; 2) results from 50 key informant (KI) interviews from state and territorial-level emergency management and public health agencies; 3) results from 14 KI interviews with deaf-serving CBOs in the San Francisco Bay Area; and 4) a pilot program evaluation of an emergency responder training serving the Deaf/HH in one urban community.

**Results:**

Results from literature review and state and territorial level KIs indicate that there is a substantive gap in emergency preparedness training on serving Deaf/HH provided by state agencies. In addition, local KI interviews with 14 deaf-serving CBOs found gaps in training within deaf-serving CBOs. These gaps have implications for preparing for and responding to all-hazards emergencies including weather-related or earthquake-related natural disasters, terrorist attacks, and nuclear-chemical disasters.

**Conclusion:**

Emergency preparedness trainings specific to responding to or promoting preparedness of the Deaf/HH is rare, even for state agency personnel, and frequently lack standardization, evaluation, or institutionalization in emergency management infrastructure. This has significant policy and research implications. Similarly, CBOs are not adequately trained to serve the needs of their constituents.

## Significance

Almost no information exists in the peer-reviewed literature about the emergency preparedness training standards and current trainings provided for Deaf and hard-of-hearing (Deaf/HH) populations. However, recent national and international disasters that required humanitarian efforts illustrate the fact that Deaf and hard-of-hearing (Deaf/HH) individuals are often not only underserved, but also particularly vulnerable, in preparing for, responding to, and recovering from emergencies [1]. In the United States, according to a landmark 2004 report by Stout, "a failing grade" was given to U.S. public warning and emergency communications systems serving the Deaf/HH post-9/11 [1]. According to the Office of Homeland Security's 2006 Nationwide Plan Review of 2,800 state and local emergency operations plans (EOPs) and related documents which included interviews with over 1,000 public safety and homeland security officials across the US, the word Deaf appeared only 8 times in their entire report [2,3].

However, FEMA's Office of Disability Integration and Coordination’s list of key concepts provide a starting point at the national level to provide guidance for ensuring people with disabilities are included in, and not left out of, the emergency management infrastructure across the US. These concepts include self-determination, no "One-Size-Fits-All," equal opportunity, inclusion, integration, physical access, equal access, effective communication, and program modifications [4].

## Background

### The Deaf/HH population

There are 48 million Deaf, deaf-blind, and hard-of-hearing (HH) people living in the United States [5]. Communication needs vary depending on level of hearing loss and cultural orientation. The diverse communication modalities in this population include American Sign Language (ASL), Signed Exact English (SEE), Pidgin Signed English (PSE), Cued Speech, lip-reading and spoken English. Recent research indicates that this population faces serious health disparities due to communication barriers and low literacy rates, including a higher risk for obesity, depression, and interpersonal violence. In particular, these communication barriers contribute to increased vulnerability in an emergency situation and present unique considerations for emergency responders [6-8]. Members of the Deaf community do not see themselves as disabled but rather as members of a linguistic minority group centered on the use of sign language, which must be taken into account when designing training programs for both emergency responders and to enhance preparedness efforts by community-based organizations (CBOs) serving the Deaf community. Cultural competence is an important consideration. It is defined by the Office of Minority Health’s National Standards for Culturally and Linguistically Appropriate Services (CLAS) in Health Care as "a set of congruent behaviors, attitudes, and policies that come together in a system, agency, or among professionals that enables effective work in cross-cultural situations [9]."

### Rationale for evaluation research

There is relatively little research or evaluation on the efficacy of preparedness efforts, particularly for those with disabilities. Given the relatively few Deaf/HH specific training programs available and the lack of evaluation of such programs, there is a need to develop best practices for emergency preparedness training for four audiences: 1) state-level emergency management and public health agencies; 2) local emergency responders; 3) Deaf-serving CBOs; and 4) Deaf community members. To date, there have been no published evaluations of trainings for emergency responders or trainings that target Deaf/HH people. In 2011, a pilot mixed methods evaluation was conducted of a training workshop for law enforcement as emergency responders for the purpose of increasing officers' cultural competency in working with Deaf and hard-of-hearing people (Deaf/HH) during domestic and sexual violence (DV/SV) emergencies [2].

### History of emergency preparedness training

To date, we have identified only fifteen training programs and disaster simulation exercises for the Deaf/HH across the country targeting emergency responders (5), certified ASL interpreters (2), deaf-blind people (1), government agencies (4) and the Deaf community (7). Some training programs target multiple audiences. These training programs range from one-time workshops to more extensive training modules (Table 1).

**Table 1:** Existing deaf preparedness training workshops and disaster simulation exercises*

**Target audience**	**Location served**	**Organization**	**Description**
**First Responders**			
First Responders	Faribault, MN	Faribault Disaster Exercise [10]	Disaster simulation video
First Responders	Riverside, CA	Riverside Disaster Exercise, California School for the Deaf at Riverside [11]	Disaster simulation video
First Responders	Gallaudet University, Washington, DC	CERT (Community Emergency Response Team), Serve DC--the Mayor's Office of Volunteerism [12]	One-time, three hour workshop (July 10–12, 2012)
First Responders	Lancaster, PA	Lancaster Volunteer Ambulance Corps [13]	Teaching emergency sign language and installing sign language apps for EMS
First Responders	Nationally	CEPIN (Community Emergency Preparedness Information Network) [14]	Training module and internet-based training in selected major cities
**Federal/state/local agencies**			
Law Enforcement	Statewide, IL	Illinois State Police Department [15]	Module placed in Illinois state police cruisers about cadet training processes.
Emergency preparedness officials	Springfield, IL	Illinois Department of Health [16]	One time conference workshop on collaborating with the Deaf/HH
Homeland Security Officials	Nationally	CEPIN [14]	Training module and internet-based training selected major cities
Law enforcement	San Francisco Bay Area, CA	DeafHope, in partnership with Oakland Police Department [2]	One-time continuing education workshop on working with the Deaf/HH in domestic violence emergencies
**Deaf/HH community**			
Deaf community	Northern California (Santa Rosa, CA)	COPE (Citizens to Prepare for Emergencies) and Sign Language People [17]	One time workshop
Deaf community	Gallaudet University, Washington, DC	CERT (Community Emergency Response Team), Serve DC--the Mayor's Office of Volunteerism [12]	One-time, three hour CERT workshop (July 10–12, 2012)
Deaf community	Rochester, NY	Red Cross Greater Rochester Chapter [18]	Safety training workshop in CPR and first aid
Deaf community	Nashville, TN	Hearing Bridges partnered with the Nashville Area Chapter of the American Red Cross and National Weather Service [19]	One-time SKYWARN Storm Spotter training on preparation for weather emergencies
Deaf community	Faribault, MN	Faribault Disaster Exercise [10]	Disaster simulation video
Deaf community	Riverside, CA	Riverside Disaster Exercise, California School for the Deaf at Riverside [11]	Disaster simulation video
Deaf community	Nationally	CEPIN [14]	Training module and internet-based training in selected major cities, including fire prevention and safety
Deaf-blind individuals	New York, NY	Helen Keller National Center [20]	12-hour curriculum
**ASL interpreters**			
ASL interpreters	Northern California (San Francisco Bay Area, Sacramento, CA)	NorCal Center on Deafness, CalEMA [21]	FAST training: Functional Assessment Shelter Team
Certified ASL interpreters	Northern California (San Francisco Bay Area, Sacramento, CA)	NorCal Center on Deafness, CalEMA [21]	Disaster Relief Interpreting Program

These types of targeted training efforts for the Deaf/HH began for the first time during the decade post-9/11 [22]. For example, in 2006, the Helen Keller National Center provided a training workshop on emergency and disaster preparation for deaf-blind people. The 12-hour multi-session curriculum focused on: disasters that affect different areas of the country; how to set up a personal support network; emergency bag and disaster kit preparation; possibilities to consider in an evacuation situation; communication with emergency responders; use of personal and emergency alert systems; rental and homeowner’s insurance; and different aspects of water and food safety [23].

In addition, in 2010, the first training of its kind in the country began for certified ASL interpreters to work as emergency responders through a partnership between California Emergency Management Agency (CalEMA) and NorCal Center on Deafness [21,24]. Three courses were offered for approximately 100 Certified ASL interpreters. According to Jordan Scott at CalEMA, when the program was being developed: “it was determined that, because of the chaotic nature of disasters, and the need for accurate and timely communication to the public in a shelter environment or during a press conference, it was important that prerequisites for the participants be established. In order to be eligible for Cal EMA’s program, the interpreter must possess a valid certification (CDI; NAD Level 3, 4 or 5; RID CI/CT; NIC Generalist, Advanced or Master Level.) In addition they must have a minimum of 10 years of community sign language interpreting experience, with 5 years of medical, law enforcement or mental health emergency interpreting experience” (Scott J, *California Emergency Management Agency*, Personal Communication, January 1, 2012). There has not been an opportunity to actually deploy any interpreters during a disaster and Cal EMA is in the process of developing an on-line refresher course so the interpreters can renew their credential status in preparation for future disasters. In general, the scope of these twelve trainings varies considerably according to target audience and geographic location. While the effectiveness and impact of these trainings still need to be evaluated, save one, the variance between them demonstrates a need for more standardization on a national level.

## Methods

We conducted a literature review, a state agency-level assessment of trainings for the Deaf/HH, a local assessment of CBO capacity, as well as a first-responder training evaluation. All interviews were conducted by trained interviewers. Informed consent was obtained from all participants for publication of this report and any accompanying images.

### Literature review

Inclusion criteria for the literature search in peer-reviewed databases included all literature or reports from the United States in English, 1990–2012. The peer-reviewed literature was searched using PubMed and Google Scholar, and grey literature on training evaluation was searched using Google, as well as by examining Community Emergency Response Team (CERT) newsletters. The following search terms were used: "Deaf CERT training," "Deaf emergency training," "Deaf disaster training," and "Deaf hurricane training," “Deaf earthquake,” “Deaf flood,” “Deaf fire,” and “Deaf all-hazards.”

### State-level agency training needs assessment

University of California, Berkeley (UCB) researchers interviewed key informants (KIs) from state and territorial level emergency management or public health agencies in order to assess emergency preparedness information and capacity to respond to the Deaf/HH during an emergency. Fifty-nine KIs (all US states, DC, and territories) were sampled, 50 KI telephone interviews were completed and 55 basic State Emergency Operation Plans (EOPs) were obtained and analyzed from agencies. The Office of Public Health Preparedness and Response (OPHPR) Extramural Research Program Offices (ERPO) and the Division of State and Local Readiness (DSLR) assisted in identifying contacts to help us obtain EOPs. State-level participants were assured of full confidentiality of their information prior to participation. For the purposes of this paper, we report analysis of three training-related interview items about departmental or other trainings attended by the KI interviewees. Other manuscripts available and in preparation will review results from remaining survey items asked of state personnel [25].

### Local-level training needs assessment

#### Sampling frame

A sample of 14 deaf-serving CBOs were selected for KI interviews based on expert opinion from the head of a deaf-serving organization in the San Francisco Bay Area in consultation with the project’s National Advisory Board (NAB) of leaders from the Deaf/HH community, a deaf graduate student researcher, and a project consultant with certification in ASL interpreting and expert knowledge of the Deaf community in Northern California. These 14 deaf-serving CBOs have on average 5,970 clients (median: 475; range: 75–60,000 clients) and of the clients served, 20-100% are Deaf or HH. For the purposes of this paper, analysis is reported for training-related survey questions only. Questions included emergency preparedness education for clients and staff, as well as client characteristics, CBO capacity to reach their constituents in an emergency, barriers and supports for the development and dissemination of preparedness materials, and partnerships with other organizations. Participants were assured of full confidentiality of their information prior to participating.

#### Instrument

We developed a semi-structured interview guide in written English for nine Deaf/HH KIs and translated the guide into ASL Gloss (a written format that approximates ASL grammar, morphology, prosody, and syntax) for five Deaf/HH KIs. A similar interview guide for Deaf women by Steinberg et al. (2002) also used ASL Gloss and defines it as standard written format used to represent ASL [26]. Before the interview, all KIs were asked how they preferred to communicate, whether it should be in spoken English, Signed Exact English (SEE), or ASL. An ASL Gloss was created for the interview guide in order to ensure that every KI interview was administered by the interviewers in a consistent format, whether in spoken English or in ASL. The ASL Gloss, developed by a Deaf graduate student researcher in conjunction with a certified ASL interpreter, was created to ensure that the instrument was linguistically and culturally appropriate.

### Local Law enforcement training evaluation

We also conducted an evaluation of a law enforcement training in Oakland, CA, to promote better response to domestic violence emergencies involving the Deaf/HH. Participants in the workshop included police officers and other law enforcement personnel, including police dispatchers. Data were collected through (1) a pre- and post-test survey [n=34] administered immediately before and after the training, and (2) two semi-structured focus groups [n=6 and n=13] with the same participants. Focus group activities occurred on the same day after two 2-hour educational outreach/training certification workshops for law enforcement personnel in the San Francisco Bay Area [2]. A trained focus group facilitator conducted the focus group. Survey items included a measurement of attitudes, including perceived *capabilities* of Deaf people, with six items such as “Deaf people can make their own life decisions” and “Deaf people can have normal one-on-one interactions on a daily basis,” and *perceived self-efficacy* when working with the Deaf/HH, with ten items such as “I feel confident I could figure out a way to communicate with Deaf people in an emergency.” Due to the dearth of instruments on Deaf preparedness, survey items were adapted from several extant instruments [27-32].

### Analytical methods

For all of these research activities, close-ended items were entered and analyzed in SPSS. We developed descriptive statistics and conducted bivariate analysis. For open-ended items, we applied qualitative coding and content analysis in Excel for identifying themes across state respondents or CBOs that were reported for contextual understanding.

## Results

The key findings include (1) a major gap in the literature related to Deaf/HH emergency preparedness, (2) evidence that the staff at state and territorial level agencies do not receive adequate cultural competency training in serving the Deaf/HH during emergencies, (3) evidence that only about 1/3 of Deaf-serving CBOs attended emergency preparedness training, only about ½ provided trainings to clients, and fewer than ½ provide preparedness education materials to clients, and (4) evidence that trainees who attended a local law enforcement training on serving the Deaf community demonstrated greater perceived self-efficacy when working with the Deaf and greater knowledge of communication and translation needs for interacting with Deaf/HH individuals following the training.

### Literature review

There is almost no literature about broader emergency preparedness communication issues for and by the Deaf/HH across various domains [2]. We could find no peer-reviewed literature specifically on emergency preparedness *training* and *evaluation* for the Deaf/HH. Zero peer-reviewed articles were found using databases including PubMed and Google Scholar.

In the “grey” unpublished literature, we were able to find and review only two documents through a Google search. They included the U.S. Fire Administration’s (2002) tip sheet for assisting Deaf/HH individuals in the event of an evacuation, and the U.S. Department of Justice’s (2009) report entitled "Victims with Disabilities: Collaborative, Multidisciplinary First Response Techniques for First Responders Called to Help Crime Victims Who Have Disabilities Trainer's Guide" [33,34]. The information in these documents aimed to help first responders communicate with the Deaf/HH in an emergency. We found no other grey literature that addressed emergency preparedness training for the Deaf/HH.

### State-level agency training needs assessment

Results indicated a significant association between a state-level KI’s familiarity with communication issues faced by the Deaf/HH and: 1) whether or not the KI’s department provides any trainings to him/her or other staff regarding how to serve the Deaf/HH populations during emergencies or disasters (p=0.02); and 2) whether or not the KI or other staff have attended any other trainings outside of their departments on serving the Deaf/HH populations during emergencies or disasters (p=0.02).

In addition, there was a significant association between a KI’s familiarity with how to make and accept relay phone calls (a critical mechanism for 2-way communication with Deaf/HH individuals during an emergency) and: 1) whether or not the KI’s department provides any trainings to him/her or other staff regarding how to serve the Deaf/HH populations during emergencies or disasters (p=0.008); or 2) whether or not the KI or other staff have attended any other trainings outside their department on serving the Deaf/HH populations during emergencies or disasters (p=0.004).

Approximately half of state KIs reported that their own department had not provided any training, however 67.3% (n=33) had attended other trainings outside of their departments on serving the Deaf population during emergencies (Tables 2 and 3).

**Table 2:** State-level Deaf/HH trainings provided

**Question**	**YES**	**NO**
***Does your department provide any training to you or other staff regarding how to serve the Deaf and Hard of Hearing populations during emergencies or disasters? (N=50)***	52% (N=26)	48% (N=24)
***Have you or other staff attended any other trainings on serving the Deaf and Hard of Hearing populations during emergencies or disasters? (N=49)***	67% (N=33)	24% (N=20)

**Table 3:** Frequency of Deaf/HH training attendance by state agencies

**Frequency of training**	**Percentage (%)**	**Responses *(N=32*)**
Annual	37.5	12
One time training	37.5	12
Attend every so often	9.4	3
As requested	6.2	2
Other^*^	9.4	3
**TOTAL**	**100**	**32**

*Includes: Training currently not available, frequency depends on funding, and does not know frequency.

### CBO data analysis

**Figure 1:**
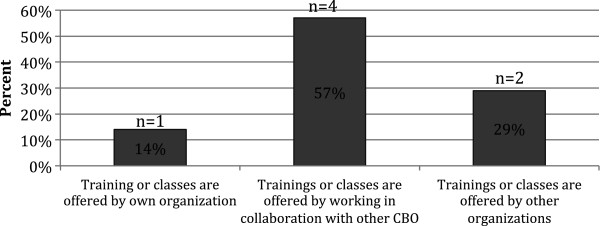
**CBO KIs providing emergency preparedness training for Deaf/HH.** Clients or Caregivers (N=7).

According to 14 KI interviews at local deaf-serving CBO's in SF Bay Area, only 36.4% of CBOs provided specific information on emergency preparedness to their clients and only 35.7% had attended trainings about serving Deaf/HH populations during emergencies or disasters at other organizations. Additionally, only half (50%) of the CBO's provided classes or trainings about emergency preparedness to clients or caregivers, and only 43% provided emergency preparedness educational materials to clients [35] (Figure 1).

Despite the fact that 50% of the deaf-serving organizations reported providing medical services, chronic disease management, and skilled nursing, only 14.3% reported providing emergency preparedness services. Therefore, in addition to a lack of training, Deaf/HH clients from these organizations are underserved in terms of emergency preparedness, evacuation services in a disaster response phase, and recovery assistance following an event. Only 21.4% of CBOs provided evacuation services and only 14.3% provided recovery assistance to their clients [36]. In addition, despite the fact that deaf-serving organizations are expected to be uniquely attuned to the literacy and functional needs of their respective constituents, written emergency preparedness materials designed for dissemination to Deaf audiences appear to be lacking. The readability of 100% (5/5) of the sample of materials collected from deaf-serving CBO’s tested above the average literacy level for Deaf/HH populations [25].

**Figure 2:**
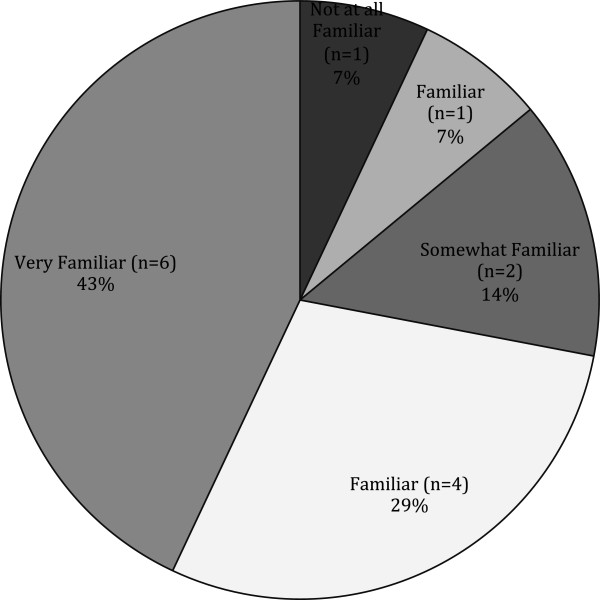
Deaf/HH-serving CBO familiarity with communication issues (N=14).

On average, KIs from deaf-serving CBOs reported a median of 12 months since attending specific training on emergency preparedness (range: 1–72 months ago). In terms of frequency of attendance, 28.6% of respondents said it was a one-time training, 28.6% said it was an annual training, and 42.9% said they attended training at some other interval (Table 4). In addition, KIs from deaf-serving CBOs had various levels of familiarity with communication issues facing their constituents in an emergency (Figure 2).

**Table 4:** Deaf-serving CBO training capacity

	**Deaf/HH-serving organizations percentage (%)**
Provide training to you or other staff about emergency preparedness (N=14)	78.6
Provide specific information on emergency preparedness for (older adult/Deaf/HH) populations (N=11)	36.4
You or staff have attended other training about serving (older adult/Deaf/HH) populations during emergencies or disasters (N=14)	35.7

Half of the KIs (50%) in deaf/HH-serving CBOs reported that the CBO provided any classes or training for clients or caregivers about emergency preparedness Figure 1. However, only a little over a third of the KIs (36.4%) reported that the CBO provided training specific to the issues that Deaf/HH people face in an emergency (Table 4).

### Evaluation of local law enforcement training

Results from both the survey (N=34) and two focus groups (total N=19) demonstrated that participants gained cultural competency skills post-training as indicated by items measuring attitudes towards the Deaf/HH, perceived better self-efficacy when working with the Deaf/HH both in a DV emergency and in a large-scale emergency, as well as by demonstrating knowledge of communication and translation needs for interacting with Deaf/HH individuals during emergencies. The attitudes subscale of the survey showed that the training had a positive impact on general attitudes towards the Deaf/HH, including *perceived self-efficacy* when working with the Deaf/HH (t(33) =-5.02, p<0.01), which, in this case, is partly a reflection of cultural competence, but not on their perception of the *capabilities of the Deaf/HH* (t(33) =-0.34, p=0.74).

However, survey participants demonstrated a lack of knowledge about federal and state-level policy and the law, which can have serious implications at the time of an emergency. For example, emergency responders encountering a Deaf/HH person at the time of an emergency may not consider securing an American Sign Language interpreter, which is required by law. Post-training, few participants were able to mention the two federal laws which protect the rights of Deaf Americans by name: The Americans with Disabilities Act (ADA) and Section 504 of the Rehabilitation Act of 1973 [2].

## Discussion

In this study, data from multiple sources: a literature review, State KI interviews, deaf-serving CBO KI interviews, and the training evaluation with first responders, all showed a critical lack of training about Deaf/HH emergency preparedness for Deaf/HH clients, CBOs, and emergency responders. Given the lack of evaluation of these types of trainings in the literature, a much more rigorous approach to assessing efficacy of the trainings is needed. The literature review indicates that there is a critical need for evaluation of trainings specific to Deaf/HH individuals in order to ensure effective training procedures and emergency preparedness programs for these populations. We also need further research on factors that promote or hinder preparedness communication capacity of agencies that serve Deaf/HH populations in disasters, deaf/HH-serving CBOs, and Deaf/HH individuals [2,25].

On a system-wide level, little information exists about national/state guidelines for emergency communication for Deaf/HH groups. However, according to FEMA's recent conference on *Promising Practices in Inclusive Emergency Management* (2011), one promising practice for training state emergency management personnel can be found in Hawaii, where people with disabilities are training emergency responders [5].

### State KIs

Despite the lack of training described by state officials interviewed, several of them brainstormed solutions and cited barriers such as cultural issues, identifying the Deaf before and during emergencies, including Deaf/HH individuals in mass notification systems, and tailoring messages to this population. They also identified systemic challenges such as a lack of training about these issues, lack of accessible infrastructure, a need for more compliance with existing legislation for mass communication, and the need for improved collaboration and partnerships with local and state-level deaf-serving organizations.

Despite the lack of training noted by state officials, several respondents discussed technological initiatives to improve access for the Deaf/HH. One respondent, referring to a question about state-level strategies for improving emergency communication for the Deaf/HH, discussed a initiative to implement a new (State) Alert and Warning System with pilot tests in two counties in which people would register their information into the system and choose how they would like to be contacted in the event of an emergency.

### Deaf-serving CBOs

Fourteen CBOs reported serving Deaf/HH clients who are minority, low-income, limited English proficiency, and low-literacy. In addition, most respondents reported serving clients who are homeless, homebound, or have limited or no transportation. These demographics indicate that training is especially important for these organizations because deaf-serving CBOs are working with individuals who have multiple risk factors before and during a disaster that may impact their safety more than many other individuals and these CBOs may have unique access to these individuals. In addition, training for deaf-serving CBOs is much needed due to the fact that these CBOs are non-profit organizations operating on very limited budgets. Especially in this economic climate, CBOs often lack resources for capacity building, which is problematic in light of the fact that Deaf/HH communities depend on local CBOs in the event of a disaster.

### Policy implications

Significant gaps in emergency preparedness training for serving the Deaf/HH exist despite the fact that in 2011 President Obama recommitted to enforcing and protecting the civil rights of people with disabilities on the 21st anniversary of the Americans with Disabilities Act (1990): "The promise of the ADA was that all Americans should have equal access and equal opportunity, including Americans with disabilities. The ADA was about independence and the freedom to make of our lives what we will. We celebrate that today, and we recommit ourselves to ending discrimination in all its forms," said President Obama [37].

This current research, coupled with more stringent enforcement of existing federal legislation, provides the kind of data and guidance that can impact national policy regarding the implementation of emergency response and planning tools that are tailor-made for the Deaf and hard-of-hearing.

The timing of this research is fortuitous. Over a decade of research indicates that input from the Deaf/HH community and from people with disabilities is increasingly being acknowledged as important and actionable, both within the research community and also in emergency management. Recent trends in emergency management include incorporating a *whole community planning* approach, which is defined by Kailes (2011) as an emerging community-oriented approach to models for practice of emergency management, and that recognizes:

“…in large scale disasters, the needs of survivors outweigh collective resources and capabilities of government . . . it really looks beyond traditional governments approach and all thinking government can solve disaster management challenges on its own. And it's really acknowledging that even small and medium sized events can be helped when government expands its reach and delivers services more efficiently by partnering with the community [38]. "

Acknowledging resource limitations in a large-scale disaster, whole community planning approaches involve: 1) non-governmental organizations, 2) businesses, and 3) government [39].

The more prepared emergency responders are in meeting the needs of Deaf/HH individuals in emergencies, the better prepared they will be in serving other vulnerable populations such as linguistic minorities. Lessons learned by addressing communication barriers faced by the Deaf/HH can be applied to 120 million Americans who also face communication barriers: low health-literate members of society (90 million) and linguistic minorities (30 million) [39,40]. Targeted preparedness training materials can be disseminated to community-based organizations, communication strategies can be targeted for unique needs of diverse communities, and training can increase the capacity of both organizations and individuals to help serve the overwhelming needs seen in many recent disasters, potentially increasing resilience in diverse communities.

#### Limitations

Limitations of the study include the small sample size inherent in US state-level analysis, the small number of Deaf-serving CBOs in our sample, and the single evaluation of a training program. However, there are only 50 states, 1 DC, and 8 territories, so this limitation is common to all state-level analysis. In addition given that deafness is a relatively low incidence disability, there is a small universe of Deaf-serving CBOs from which to choose. For the evaluation study, only one of the instruments that we adapted for use was peer reviewed due to the fact that there was a lack of available instruments. Our instruments were also newly created and pilot-tested with experts in, and affiliated with, the Deaf community and were created out of necessity because there were none available. Future researchers could attempt to replicate findings. Findings may have applicability to other populations with communication barriers such as limited-English proficiency populations.

## Conclusion

### Future directions

Our research indicates the need for: 1) increased accessibility and involvement of the Deaf/HH in training and exercises with guidance from state personnel; 2) required cultural competence training for first responders in order for them to better understand the diversity within this population (Deaf/deaf-blind/hard-of-hearing/late deafened) and improved emergency responder communication approaches; 3) standardized guidelines for CBOs to participate in local emergency and preparedness planning and exercises as fully as possible; and 4) development and dissemination of national guidelines about functional and access needs specific to the Deaf/HH population to emergency preparedness and response trainers across the nation [37]. Establishing a national taskforce is a critical first step in the development of appropriate guidelines.

Since 9/11, important first steps have been taken to train emergency responders, Deaf/HH individuals, and sign-language interpreters to prepare for emergencies or disasters. However despite the limitations of our predominantly qualitative study, our research indicates that trainings specific for Deaf/HH are rare, even for state agency personnel, frequently lack standardization or evaluation, have not been institutionalized in the emergency management infrastructure, and thus there is no consistent curriculum across agencies. Programs on which we found information had few quality control or quality improvement measures and demonstrate a lack of coordination of efforts across agencies and organizations which are providing the trainings. On a policy level, there needs to be more mainstreaming of the needs of the Deaf/HH in emergency training and operations. In the long term, increasing awareness among first responders, emergency management agencies, and deaf-serving CBOs about the needs of Deaf before, during, and after disasters may lead to higher-level policy changes and improved outcomes for Deaf individuals.

Given the enormous diversity within the Deaf/HH population, developing, implementing, and evaluating emergency preparedness training cannot be achieved without forming alliances between agencies charged with emergency response and the Deaf/HH community, including getting input from experts in the emergency preparedness field who are Deaf/HH.

Our fundamental recommendation regarding accessible emergency preparedness communication design is to ensure close participation of its intended beneficiaries as well as those involved in the communication dissemination and evaluation. All-hazards emergency preparedness communication challenges can be mitigated by a participatory design process, or a co-production of knowledge between the lived experience of Deaf/HH people, EMS system practitioners, and emergency preparedness researchers [25,41,42].

## Abbreviations

ADA: The Americans with Disabilities Act; ARC: American Red Cross; ASL: American Sign Language; Cal EMA: California Emergency Management Agency; CBO: Community Based Organization; CDI: Certified Deaf Interpreter; CEPIN: Community Emergency Preparedness Information Network; CERT: Community Emergency Response Team; CLAS: Culturally and Linguistically Appropriate Services; COPE: Citizens to Prepare for Emergencies; CSDR: California School for the Deaf at Riverside; DCARA: Deaf Counseling Advocacy and Referral Agency; Deaf/HH: Deaf and hard-of-hearing; DRI: Disaster Relief Interpreting; DSLR: Division of State and Local Readiness; DV: Domestic Violence; EMS: Emergency Medical Services; EOP: Emergency Operations Plan; ERPO: Extramural Research Program Offices; FAST: Functional Assessment Shelter Team; FEMA: Federal Emergency Management Agency; HRA: Health Research for Action; KI: Key Investigator; NAB: National Advisory Board; NAD: National Association for the Deaf; NIC: National Interpreter Certification; NWS: National Weather Service; OPHPR: The Office of Public Health Preparedness and Response; PERRC: Preparedness and Emergency Response Research Center; PSE: Pidgin Signed English; RID CI/CT: Registry of Interpreters for the Deaf Certificate of Interpretation/Certificate of Transliteration; SEE: Signed Exact English; SV: Sexual Violence; UCB: University of California, Berkeley

## Competing interests

The authors declare that they have no competing interests.

## Authors’ contributions

AE, SI, WT, and LN conceived the study, AE, SI, WT, LN designed the study. AE, SI, WT, and DD obtained, prepared, and managed the data, performed the qualitative and quantitative analyses and AE conducted literature review. AE wrote the initial manuscript, and SI helped to draft the manuscript. All authors interpreted the findings and approved the final manuscript.

## Authors’ information

Alina Engelman, DrPH, MPH, received her doctoral degree in Public Health at UC Berkeley. While completing her MPH in Global Health at Yale University, she worked in Kenya on a program evaluation of HIV/AIDS services for the deaf using community-based participatory research (CBPR) techniques. In addition to her role as a research associate on UCB PERRC's "All-Hazards Communication to Improve the Resilience of Vulnerable Populations” she was also the recipient of a Health Policy & Health Systems Research pilot project award from the Center for Infectious Disease and Emergency Readiness at UC Berkeley.

Susan L. Ivey, MD, MHSA, is Director of Research at HRA and Associate Professor, adjunct, in the Department of Community Health and Human Development at UC Berkeley School of Public Health. Dr. Ivey is board-certified in family medicine and practices medicine part-time with the City of Berkeley Public Health division, including on preparedness planning.

Winston Tseng, PhD, is Research Sociologist in the Department of Community Health and Human Development at UC Berkeley School of Public Health. He has a background in medical sociology and extensive community-based participatory research (CBPR) experience with diverse and vulnerable populations.

Donna Dahrouge, MPH, is a Research Analyst. She is the project manager for Project 1 of the Preparedness and Emergency Response Research Center (PERRC) funded by the CDC.

Jim Brune, BA, is the Executive Director of Deaf Counseling Advocacy and Referral Agency (DCARA), which is a non-profit, community-based social service agency serving the Deaf community in the San Francisco Bay Area.

Linda Neuhauser, DrPH, is Clinical Professor in the Department of Community Health and Human Development at UC Berkeley and Co-Principal Investigator of the Health Research for Action center. She focuses on research translation and has a special interest in collaborative design and evaluation of mass communication that meets people's literacy, language, cultural, disability, and other needs.

## Pre-publication history

The pre-publication history for this paper can be accessed here:

http://www.biomedcentral.com/1472-6963/13/84/prepub
